# Human Serum Amyloid A3 (SAA3) Protein, Expressed as a Fusion Protein with SAA2, Binds the Oxidized Low Density Lipoprotein Receptor

**DOI:** 10.1371/journal.pone.0118835

**Published:** 2015-03-04

**Authors:** Takeshi Tomita, Katsuaki Ieguchi, Tatsuya Sawamura, Yoshiro Maru

**Affiliations:** 1 Department of Pharmacology, Tokyo Women’s Medical University, Tokyo, Japan; 2 Department of Vascular Physiology, National Cerebral and Cardiovascular Center, Suita, Osaka, Japan; The Research Institute for Children, UNITED STATES

## Abstract

Serum amyloid A3 (SAA3) possesses characteristics distinct from the other serum amyloid A isoforms, SAA1, SAA2, and SAA4. High density lipoprotein contains the latter three isoforms, but not SAA3. The expression of mouse SAA3 (mSAA3) is known to be up-regulated extrahepatically in inflammatory responses, and acts as an endogenous ligand for the toll-like receptor 4/MD-2 complex. We previously reported that mSAA3 plays an important role in facilitating tumor metastasis by attracting circulating tumor cells and enhancing hyperpermeability in the lungs. On the other hand, human SAA3 (hSAA3) has long been regarded as a pseudogene, which is in contrast to the abundant expression levels of the other isoforms. Although the nucleotide sequence of hSAA3 is very similar to that of the other SAAs, a single oligonucleotide insertion in exon 2 causes a frame-shift to generate a unique amino acid sequence. In the present study, we identified that hSAA3 was transcribed in the hSAA2-SAA3 fusion transcripts of several human cell lines. In the fusion transcript, hSAA2 exon 3 was connected to hSAA3 exon 1 or hSAA3 exon 2, located approximately 130kb downstream from hSAA2 exon 3 in the genome, which suggested that it is produced by alternative splicing. Furthermore, we succeeded in detecting and isolating hSAA3 protein for the first time by an immunoprecipitation-enzyme linked immune assay system using monoclonal and polyclonal antibodies that recognize the hSAA3 unique amino acid sequence. We also demonstrated that hSAA3 bound oxidized low density lipoprotein receptor (oxLDL receptor, LOX-1) and elevated the phosphorylation of ERK, the intracellular MAP-kinase signaling protein.

## Introduction

Serum amyloid A (SAA) is a very important protein that is markedly up-regulated in acute phase inflammatory responses. Four SAA isoforms have been identified at the protein level in the mouse, and were shown to be approximately 10 kDa in size after the signal peptide was removed [1–4]. Mouse SAA1 and SAA2 are well-known to be expressed in hepatocytes, whereas mSAA3 is expressed extrahepatically in cells including adipocytes, epithelial cells, and endothelial cells [5–7]. While these three isoforms are inducible isoforms, mSAA4 is constitutively expressed in hepatocytes. Moreover, in mouse, SAA1, SAA2, and SAA4 were shown to be components of high density lipoprotein, while SAA3 was not [8]. SAA1 and SAA2 can also be expressed extrahepatically. Human aortic smooth muscle cells synthesize SAA1 and SAA2 when stimulated with dexamethasone and IL-1β [9]. Mouse SAA3 is known to play an important role in tumor metastasis. Our previous studies revealed the secretion of mSAA3 in lung endothelial cells or epithelial cells to be a pivotal step in the formation of the pre-metastatic phase [5, 7, 10]. The injection of a mSAA3 neutralizing antibody effectively prevented tumor metastasis to the lungs in tumor-bearing mouse model assays. In addition, biochemical analyses revealed that mSAA3 is an endogenous ligand for the toll like receptor 4 (TLR4)/MD-2 complex, the epicenter for the innate immune system [11], and also that the S100A8-SAA3-TLR4 cascade is responsible for myeloid-derived cell recruitment in the lungs and attracts circulating tumor cells to the lungs. Human SAA1, hSAA2, and hSAA4 proteins are similar to the mouse protein equivalents, while hSAA3 has a frame shift in the open reading frame due to the insertion of a single nucleotide, which completely changes the translated amino acid sequence, resulting in the generation of a peptide (60 amino acids). Thus, hSAA3 is considered to be a pseudogene and is classified as a non-coding RNA in the genome database [12]. The hSAA2-SAA3 readthroughs were found in several human cell lines, although their expression levels were extremely low. Yeast two hybrid assays pointed out that LOX-1 (also known as OLR1) was a potential endogenous receptor for hSAA3 domain. LOX-1 is a major receptor for oxidized low-density lipoproteins in endothelial cells, such as human umbilical vascular endothelial cells (HUVEC) [13]. Intracelluar signaling analysis found that hSAA3 signaling might be related to ERK phosphorylation, similar to the previous observations that SAA protein bound CLA-1 or CD36 to induce ERK and p38 mitogen-activated protein kinases [14, 15]. Human SAA3 has not been studied in detail; only one previous study, which determined the exon structure and its nucleotide sequence empirically, has been published to date [16]. In the present study, we determined 5’ region of hSAA3 transcripts for the first time to find out that hSAA3 is expressed as a part of the hSAA2-SAA3 readthrough, encoding either the hSAA2-SAA3 fusion protein or truncated hSAA2 in the first cistron and a short peptide in the second cistron.

## Materials and Methods

### Cell culture

Two different human lung carcinoma cell lines, LU65 and LU99, and human breast adenocarcinoma cell line MCF7, were obtained from the RIKEN Cell Bank. HUVEC, human lung carcinoma cell line H292, and human ductal breast epithelial tumor cell line T47D were from American Type Culture Collection (ATCC). HUVEC was maintained in endothelial cell culture media (Life Technologies) with endothelial cell growth supplement (Millipore). LU65 and LU99 cells were cultured in DMEM/F-12 + 10% FBS. MCF7, T47D, and H292 cells were in RPMI 1640 + 10% FBS. CHO cells transfected with the control vector or human LOX-1 tetracycline-inducible expression vector were cultured in F12 (Wako Pure Chemical). CHO cells were treated with serum-free medium containing 1 mg/L doxycycline for 16 h prior to the experiments. Cells were stimulated with 100 nM dexamethasone (Sigma-Aldrich, D4902), 10 ng/ml human IL-1β (Miltenyi Biotec, Auburn, CA USA), and 10 ng/ml IL-6 (Miltenyi Biotec), or 1 μg/ml LPS (Sigma-Aldrich, L2880) for 6 h for qPCR analyses or for 3 days for protein analysis.

### Antibodies and proteins

Recombinant truncated LOX-1 (61–273 amino acids, extracellular domain) with a hexa-histidine tag (C-terminal) was over-expressed in HEK293T cells and purified by affinity resin for the histidine tag. Recombinant CD36 (hexa-histidine tag at C-terminal) was purchased from GE Healthcare (10752-H08H). Recombinant hSAA1 was obtained from Peprotech (Rocky Hill, NJ) The hSAA2-SAA3 fusion protein with DYKDDDDK tag at the C-terminal end was over-expressed in HEK293T cells (15 cm dish x 1,000) and purified by anti-DYKDDDDK agarose column (1 ml, Wako Pure Chemicals) and further purified with DEAE Sepharose FF (1 ml, GE Healthcare). SDS-PAGE analysis of the purified hSAA2-SAA3 gave one extra band at 30–40 kDa other than a band (10–13 kDa) of target molecule. Synthesized peptides for hSAA3, C-terminal 44 mer, 99% purity) and scrambled 44 mer (96% purity) were purchased from Scrum Inc. (Tokyo, Japan) and 26 mer (97% purity) was obtained from Operon Biotechnology (Tokyo, Japan), respectively. The amino acid sequence of scrambled-44 mer is as follows; DCKWKEQMTTNIMWAASPFASYAKLQLKYFKGQLGLRGLGSEDK. The 42 mer hSAA3 peptide (60% purity, purchased from Life Technologies, Carlsbad, CA) was used as an antigen to generate the mouse monoclonal antibody and rabbit polyclonal antibody. To prepare the monoclonal antibody, we screened hybridoma cell lines produced by the fusion of immunized mouse spleen cells and myeloma cells. A cell line capable of continuously producing the monoclonal antibody was finally isolated. The monoclonal antibody for hSAA3 was purified from hybridoma conditioned medium. To prepare the polyclonal antibody, rabbit antisera were purified with a Protein A column and affinity column immobilizing the hSAA3 C-terminal 26 mer peptide. Purified antibodies were validated by western blotting.

### Preparation of proteins fused to gluthathione S-Transferase (GST)

PCR products for hSAA2, hSAA2-SAA3a, hSAA3 (encoding C-term 42 mer), and mouse SAA3 were cloned into pGEX vector (GE healthcare). Each construct was transformed into E. coli (BL21 strain, Lucigen, Middleton, WI). GST-fusion proteins were purified from the E.coli lysate by using Glutathione Sepharose (GE Healthcare). Purified proteins were loaded on a 12% polyacrylamide gel and electrotransferred onto a polyvinylidene difluoride membrane. Then, the membrane was probed with the hSAA3 antibody, hSAA2 antibody (Sigma-Aldrich, SAB1401350), or GST antibody (MBL Co. Ltd. (Nagoya, Japan), PM013–7). To prepare expression plasmids, PCR reactions were performed using the following primer sets; mSAA3, 5’-GAATTCAGATGGGTCCAGTTCATGAAAGAA-3’ and 5’-CTCGAGTCAGTATCTTTTAGGCAGG-3’; hSAA3, GGATCCCAAGGATGGTTAACATTCCTCAAG-3’ and 5’-CTCGAGTCATAGTTCCCCCAAG-3’; hSAA2, 5’-GGATCCAGCTTCTTTTCGTTCCTTGGCGAG-3’ and 5’-CTCGAGTTACAGTTCAGCTGAAAATAAACT-3’; hSAA2-SAA3a, 5’-GGATCCAGCTTCTTTTCGTTCCTTGGCGAG-3’ and 5’-CTCGAGTCATAGTTCCCCCAAG-3’. The PCR products were cloned into EcoRI-XhoI or BamHI-XhoI site of the pGEX vector.

### RT-PCR/qPCR

Total RNAs were isolated from cells using Trizol (Life Technologies), and further purified with RNeasy Plus (QIAGEN) or oligo dT magnetic beads (Fast track MAG, Life Technologies). Purified RNA was used to synthesize cDNA. The oligo dT or gene-specific primer for hSAA3 (5’-TTCAGTAACTGTAGGTCAAC-3’) was used in the reactions of reverse transcriptase (Primescript II (Takara) or Superscript III (GE Healthcare)). The primers used for PCR analyses were as follows; hSAA2ex2F#1[5’-GCCTACTCTGACATGAGAGAAGCCAA-3’ (hSAA2 exon 2)], hSAA3ex1F#1 [5’-ATGAAGCTCTCCACTGGCATCATTTTC-3’ (hSAA3 exon 1)], hSAA3ex2R#2 [5’-GCTCTGCTCACTCATTCCTGGCAACA-3’ (hSAA3 exon 2)], hSAA3ex2R#3 [5’-GCAATCCTCTGCATGGTCTCCTGTGA-3’ (hSAA3 exon 2)]. The PCR conditions were 94°C followed by 40 cycles of 98°C for 10s and 68°C for 60s. Quantitative PCR primers and probe sets were described below; hSAA2: 5’-GCAGAAGTGATCAGCAATGCCAGA-3’, 5’-ATTTATTGGCAGCCTGATCGGCCA-3’, and 5’-ATATCCAGAGACTCACAGGCCATGGT-3’(probe), (hSAA2 exon 3)-(hSAA3 exon1): (to detect hSAA2-SAA3a), 5’-TGTGGAGAGCCTACTCTGACATGA-3’, 5’-TTAACCATCCTTGGCTGCTGACAC-3’, and 5’-AGGATGAAGCTCTCCACTGGCATCAT-3’(probe), (hSAA2 exon 3)-(hSAA3 exon2): (to detect hSAA2-SAA3b), 5’-CCGCAGAAGTGATCAGGGACTAAA-3’, 5’-CTGCTCACTCATTCCTGGCAACAT-3’, and 5’-TGTCTGGGCTACAGAAGTGATCAGGT-3’(probe). To quantify β-actin and (hSAA3 exon 1)-(hSAA3 exon 2), primer/probe sets were purchased from Life Technologies (Cat. No. Hs01060665_g1 and Hs01380779_m1). All primer sets are designed to detect the exon-exon junction. Quantitative PCR analysis was performed using the Taqman Fast Advanced Master Mixture (Applied Biosystems, Carlsbad CA, USA) and StepOnePlus real time PCR system (Applied Biosystems). Gene expression levels were calculated from Ct values using the corresponding isolated DNA and its serial dilutions as a standard. Please see supporting information (S1 Table and S1 Fig.) in details.

### Rapid amplification of cDNA Ends (RACE)

The SMARTer RACE cDNA Amplification Kit (Takara) was used to determine the 5’ region nucleotide sequences of the transcripts. Gene-specific primer sets [exon1-R primer; 5’-CATGTCTTTAGTCCCTTGGCCAGCTG-3’ (hSAA3ex1R#1), hSAA3ex2R#2, and hSAA3ex2R#3] were used.

### Enzyme-linked immunosorbent assay (ELISA)

Rabbit polyclonal anti-hSAA3 and mouse monoclonal anti-hSAA3 were treated with pepsin (Roche Applied Science) and ficin (Wako Pure Chemical), respectively. Both antibodies were subjected to a gel filtration column (superdex 200pg, 1.6 mm x 600 mm) to purify the F(ab)_2_’fragments. The polyclonal F(ab)_2_’ fragment was labeled with biotin (EZ-link Sulfo-NHS-Biotinylation Kit, Thermo Scientific) prior to use. The monoclonal F(ab)_2_’ fragment was used for a 96-well ELISA plate coating (2 μg/ml PBS, overnight, 4°C). The following day, each well was blocked with blocking buffer (Starting Block Blocking Buffer, Thermo Scientific) and samples and standards were applied. The plate was incubated for 1 h at RT and washed with PBS-0.1% Tween20. Biotinylated polyclonal F(ab)_2_’ (1 μg/ml PBS-0.1% Tween20–1% BSA) was placed in each well and left to stand for 1 h. After seven washes with PBS-0.1% Tween20, wells were incubated with Neutravidin-HRP (Thermo Scientific) for 1 h. After pre-incubation washing protocol were repeated followed by colorimetric detection using TMB substrate (BD Bioscience) solution. Hydrochloric acid (3 M) was used as a quenching solution. Optical absorbance was recorded on a plate reader (Thermo Scientific, Multiscan G0) at 450 nm and 650 nm. Serial dilutions of the synthetic hSAA3 peptide (C-terminal 26 mer, 10–100,000 pg/ml) were used as the standard.

Purified recombinant human LOX-1 protein (1 μg/ml) was coated in each well for the binding assay. After blocking with PBS-10% FBS, various concentrations (10–2,000 ng/ml) of the synthetic hSAA3 peptide diluted in PBS-0.05% Tween20–0.5% Triton X-100 were applied to the wells (0.1 ml per well in a 96 well plate). The wells were further washed with PBS-0.05% Tween20 five times and the hSAA3 peptides retained on the wells were quantitated using the monoclonal anti-hSAA3-HRP conjugate and TMB substrate.

### Cytokine secretion analysis

The basic procedures of IL-8 assay were slightly modified from the previous publication [14]. Blank vector or human CD36 expression vector cloned into pcDNA6.2 was transfected into HEK293T cells with transfection reagent (Hilymax, Dojindo). The following day, culture medium was switched to DMEM-0.2% BSA and further cultured at 37°C, 20 h. IL-8 levels in the culture supernatants were analyzed by an ELISA system (R&D Systems Inc.), and cells were lysed with cell lysis buffer (50 mM HEPES, pH 7.9, 150 mM NaCl, 0.5% Triton X-100, 0.5% Sodium cholate) to determine its protein concentration (BCA protein assay reagent, Thermo Scientific). To confirm the over-expression of CD36, cells with CD36 expression plasmid were analyzed by a flow cytometer (FC-500, Beckman Coulter) after probing with a monoclonal CD36 antibody (1 μg/ml, FA6.152, Beckman Coulter) followed by an anti-mouse IgG-alexa 488 (0.7 μg/ml, GE Healthcare).

### Fluor-labeled ligand uptake

Recombinant hSAA1 protein, hSAA3 C-terminal 44 mer peptide, and scrambled 44 mer peptide were conjugated with Hilyte fluor 647, using a labeling kit (Dojindo LK15). HeLa cells were incubated with fluor-labeled ligands or 3, 3’-dioctadecylindocarbocyanine (DiI)-oxLDL at 37°C for 2 h. Next, cells were washed with PBS and detached with Cell dissociation solution (Sigma-Aldrich, C5914) for the following analysis by using a flow cytometer.

### Immunoaffinity column purification

The rabbit polyclonal antibody (anti-hSAA3) was covalently immobilized to hydrazine beads (Affi-Gel Hz Immunoaffinity Kit, BioRad). The beads were packed in a glass column and washed with PBS. The cell culture medium was loaded on the column and washed with PBS and PBS-1 M NaCl. After washing, proteins were eluted with 0.1 M glycine, pH 3.0, 0.5% Triton X-100, and 0.5 M NaCl. The effluent was neutralized before proceeding to assays.

### Yeast two hybrid assay

We used MACHMAKER two hybrid system 3 (Takara). In this assay, nucleotides encoding (hSAA3 exon 1)-(hSAA3 exon 2) to generate the hSAA3 C-terminal 61 mer polypeptide were incorporated into a pGBKT7 vector to use a fusion protein with GAL4 DNA-BD as bait, and the human placenta cDNA library cloned into a pACT2 vector was used as prey. Detailed screening procedures are described in the manual provided by the manufacturer. To prepare bait vectors, PCR reactions were performed using the following primer sets; hSAA3, 5’-GAATTCACCATGAAGCTCTCCACTG-3’ and 5’-GTCGACTCATAGTTCCCCCAAGCAT-3’; m SAA3, 5’-GAATTCATGAAGCCTTCCATTGCCATCATTCT-3’ and 5’-CTGCAGTCAGTATCTTTTAGGCAGGCCAGCAG-3’. The PCR products were cloned into the EcoRI-PstI site of the pGBKT7 vector.

PCR primer sets (Human LOX-1, 5’-GGATCCAAATGTCCCAGGTGTCTGACCT-3’ and 5’-CTCGAGTCACTGTGCTCTTAGGTTT-3’; Mouse LOX-1, 5’-GGATCCAAATGCGCCAGGTATCTGACCT-3’ and 5’-CTCGAGCTAAATTTGCAAATGATTTGTCTT-3’) were used to prepare prey vectors. The PCR products were cloned into the BamHI-XhoI site of the pGADT7 vector.

### Surface plasmon resonance (SPR) analysis

Detailed procedures were described in our previous paper [11]. Briefly, recombinant CD36 protein was immobilized on a GLM sensor chip of the ProteON XPR36 protein interaction array system (Bio-Rad, Hercules, CA). The hSAA3 44 mer peptide or scrambled 44 mer peptide diluted with PBS-Tween20 buffer (Bio-Rad) was applied on the chip with a flow rate of 50 μl/min. Sensorgrams were analyzed by software equipped on the system.

### Statistical analysis

All data were expressed as mean ± standard deviation and analyzed using Mann-Whitney U test or Kruskal-Wallis test followed by a post-hoc comparison. All statistical hypothesis testing results and additional data tables (S2 Table, S2 Fig., and S3 Table, S3 Fig.) were included in the supporting information.

## Results

**Fig 1:**
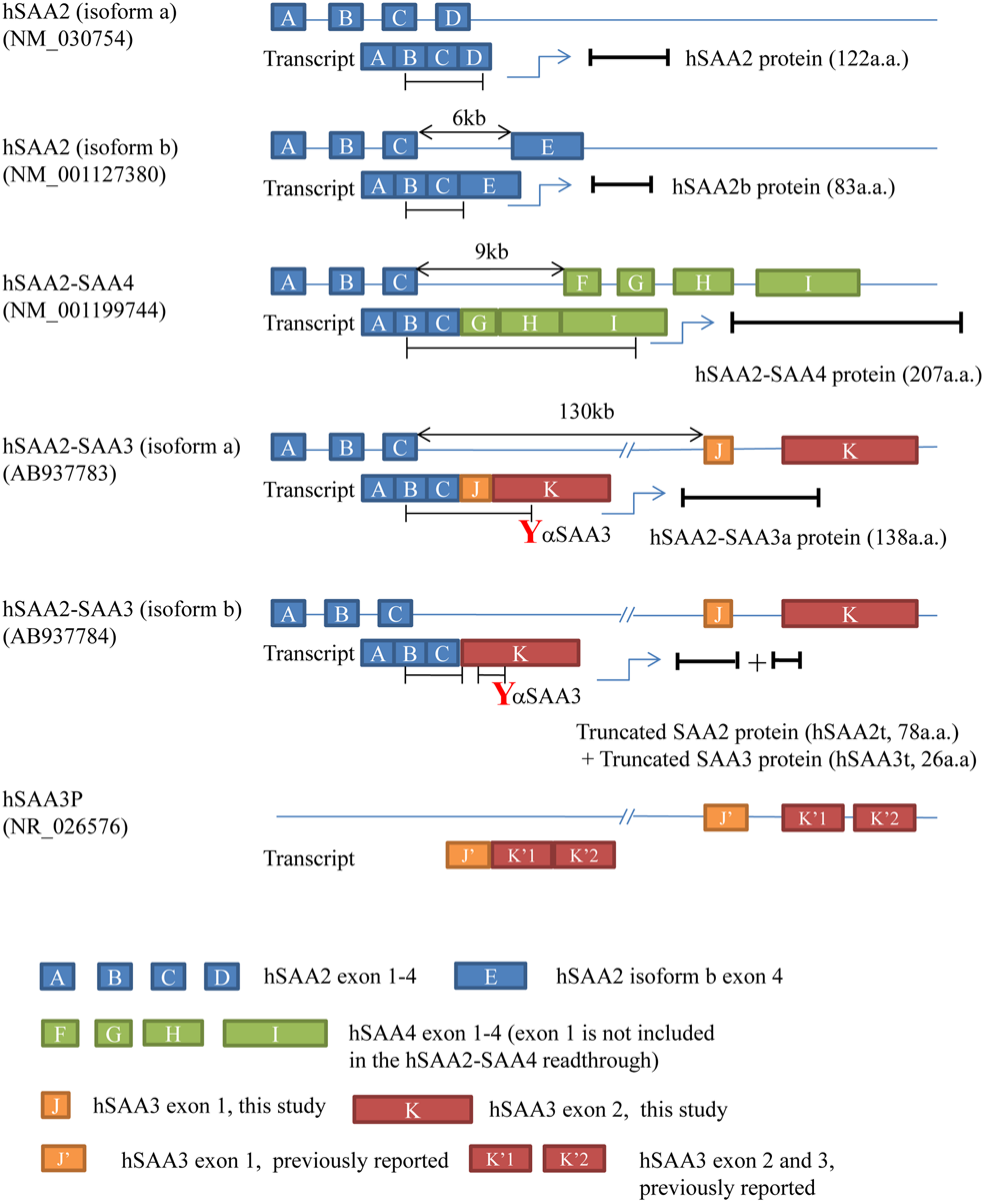
Structures of hSAA2-SAA3 readthroughs. A-K stand for exons in the hSAA2, hSAA3, or hSAA4 gene. Human SAA3 exon 1 (J) identified in the present study was 52 bp shorter than that previously reported (J’). Human SAA3 exon 2 (K) in the present study contained intron between hSAA3 exon 2 (K’1) and exon 3 (K’2). The 5’ edge of (K) was identical to that of (K’1), as was the 3’ edge of (K) to that of (K’2). The bars under the transcripts represent open reading frames. As displayed in the figure, our hSAA3 antibodies recognize the C-terminal region of hSAA3.

All hSAA3 transcripts obtained from the cell lines used in this study was the hSAA2-SAA3 readthrough (Fig. 1). The first three hSAA2 exons (hSAA2 exon 1)-(hSAA2 exon 2)-(hSAA2 exon 3) fused with either the (hSAA3 exon 1)-(hSAA3 exon 2) fragment (hSAA2-SAA3a; AB937783) or (hSAA3 exon 2) alone (hSAA2-SAA3b; AB937784). The hSAA3 exon 1 determined in this study was different from that in the genome database. Human SAA3 exon 1 is registered as 147 bp in size in the database. We determined that 52 bp of the 5’ side of hSAA3 exon 1 was missing in the readthrough. Determinations of the 5’- upstream sequences of the hSAA3 transcripts were executed by the 5’ RACE method. The oligo dT based primer was used to synthesize cDNA in these experiments; therefore, it is highly likely that these hSAA2-SAA3 readthroughs were polyA+ mRNA. We confirmed that cDNA prepared form oligo-dT magnetic beads-enriched RNA contained the hSAA2-SAA3 readthrough. In addition, hSAA3 exon 2 identified in this study held an intron between hSAA3 exon 2 and hSAA3 exon 3 that were assigned in the previous study. Because the splicing site of the 5’ side of hSAA3 exon 2 remained unchanged, the expected size of the newly found hSAA3 exon 2 should be 211 + 321 + 284 = 816 bp.

**Fig 2:**
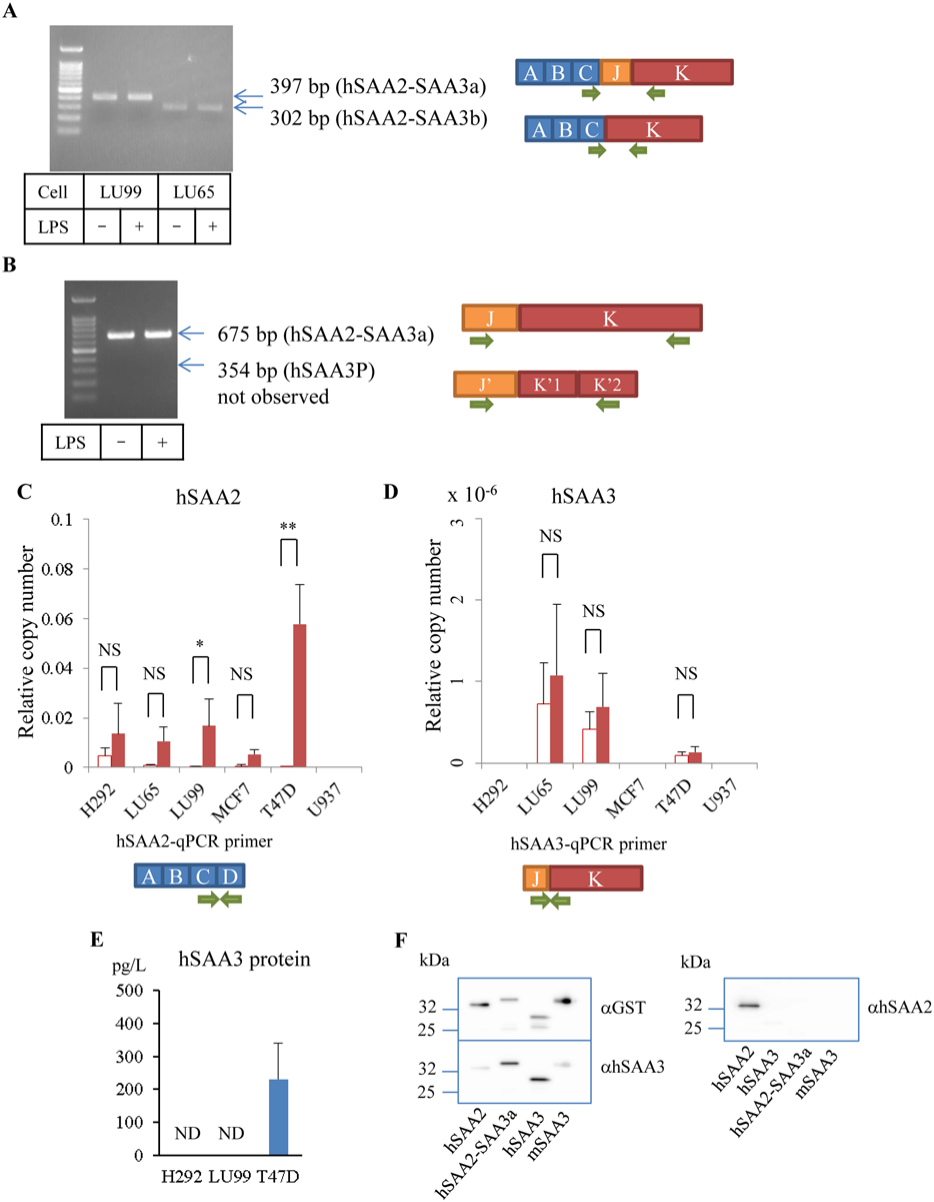
PCR detection of hSAA2-SAA3 readthroughs. (A) PCR with the hSAA2-SAA3-specific primer set. (B) PCR with the hSAA3-specific primer set. Complimentary DNAs were synthesized using a gene-specific primer that binds hSAA3 exon 2. The PCR products were subjected to DNA sequencing analyses to confirm that these bands were hSAA2-SAA3 readthrough origin (three independent experiments). (C) hSAA2 expression levels normalized to β-actin. *P = 0.0096, **P = 0.0001. (D) hSAA3 expression levels normalized to β-actin. Open bars and filled bars represent without/with IL-1β-IL-6-dexamethazone stimulation, respectively (four independent experiments). NS = not significant (P > 0.05). (E) hSAA3 concentrations determined by Immunoprecipitation (IP)-ELISA. Culture media were passed through the immunoaffiinity column, and the effluents were assayed (four independent experiments). ND = not detected. (F) Western blot analysis of hSAA3 antibody. Purified hSAA2, hSAA2-SAA3a, hSAA3, and mSAA3 proteins fused to GST were blotted onto a membrane and detected with GST (left top), hSAA3 (left bottom), and hSAA2 (right) antibodies.

RT-PCR using the gene-specific primer for first strand synthesis clearly isolated transcripts containing the hSAA3 domain. Agarose gel images showed two types of PCR products amplified with the forward primer in hSAA2 exon 3 and reverse primer in hSAA3 exon 2 (Fig. 2A), which correspond hSAA2-SAA3a and b transcripts. Oligonucleotide sequence analyses confirmed that the PCR products were hSAA2-SAA3 readthroughs. The hSAA2-SAA3a transcript gave a hSAA2-SAA3 fusion protein, which was composed of 76 amino acids from the hSAA2 N-terminal region and 62 amino acids from the entire hSAA3 region. The first 18 residues of the hSAA2 N-terminal region would be removed as the signal peptide to form a mature protein. On the other hand, the hSAA2-SAA3b transcript gave two peptides due to the presence of a stop codon in hSAA3 exon 2, the hSAA2 N-terminal region (hSAA2t, 78 a.a.), and truncated hSAA3 (hSAA3t, 26 a.a.). The latter short peptide, identical to the C-terminal region of hSAA2-SAA3a, should be translated as the second cistron, and its expression efficiency was reduced up to 1% of the first cistron [17]. We confirmed this ratio using an *in vitro* translation system and ELISA (data not shown). The PCR reaction to amplify (hSAA3 exon 1)-(hSAA3 exon 2) detected a 675 bp fragment, but failed to detect a 354 bp fragment (Fig. 2B). The hSAA3 transcripts identified in the present study contained the entire intron between that formerly assigned as hSAA3 exon 2 and hSAA3 exon 3. The presence of the intron did not affect the deduced amino acid sequences of the readthroughs because the stop codon in hSAA3 exon 2 was located before the intron. Oligo dT beads purification of RNA-based or oligo dT-primer based reverse transcription did not alter the PCR product pattern, which indicated that hSAA2-SAA3 readthroughs are polyA+ transcripts. LPS stimulation did not induce any modifications in the PCR results. In addition, our qPCR analyses (described below) confirmed that LPS stimulation did not upregulate expression of hSAA3 containing transcripts.

To count the copy number of these transcripts, we conducted quantitative PCR analysis. Fig. 2 present the relative copy numbers, normalized to β-actin, of several cell lines. The hSAA2 copy number markedly increased, particularly in T47D cells, with statistical significance when cells were treated with IL-1β-IL-6-Dexamethazone (Fig. 2C); however, the hSAA3 copy number probed by a qPCR primer set to detect the (hSAA3 exon 1)-(hSAA3 exon 2) junction was maintained at a very low level (Fig. 2D). In this case IL-1β-IL-6-Dexamethazone stimulation did not yield apparent increase in the expression levels of hSAA3 containing transcripts. Other types of qPCR primer sets to amplify (hSAA2 exon 3)-(hSAA3 exon 1) and (hSAA2 exon 3)-(hSAA3 exon 2) failed to quantitate transcripts, which suggested that the expression levels of these transcripts were extremely low.

Although hSAA3 gene expression levels were very low, we succeeded in isolating the hSAA3 protein. A large amount of conditioned media (3L) from cell lines stimulated with IL-1β-IL-6-Dexamethazone for 3 days were subjected to a polyclonal hSAA3 antibody-immobilized column and the effluent was analyzed by sandwich ELISA. Only T47D culture medium was positive (231 ± 112 pg/L, Fig. 2E) for the hSAA3 protein, while the other two cell lines (LU99, H292) were lower than the detectable level. Although this assay does not establish whether it is fused with hSAA2, there is no doubt regarding the existence of the hSAA3 protein. The antibodies used in this study recognized the hSAA3 C-terminal unique sequence. Since this ELISA used a biotin-labeled antibody and neutravidin-HRP conjugate, antibody leakage from the column does not interfere with the assay.

To confirm specificity of the hSAA3 antibody, we demonstrated western blots using mSAA3, hSAA3, hSAA2-SAA3a, and hSAA2 proteins fused to GST (Fig. 2F). Anti-GST probing visualized precise mobility of these fusion proteins in the gel (left top panel). After stripping process, the membrane was re-probed with anti-hSAA3 to give clear contrast between hSAA3 and hSAA2-SAA3a, and others (left lower panel). In contrast, hSAA2-SAA3a was not detected by anti-hSAA2 probing (right panel), suggesting that epitope of this antibody was located at the C-terminal region of hSAA2.

**Fig 3:**
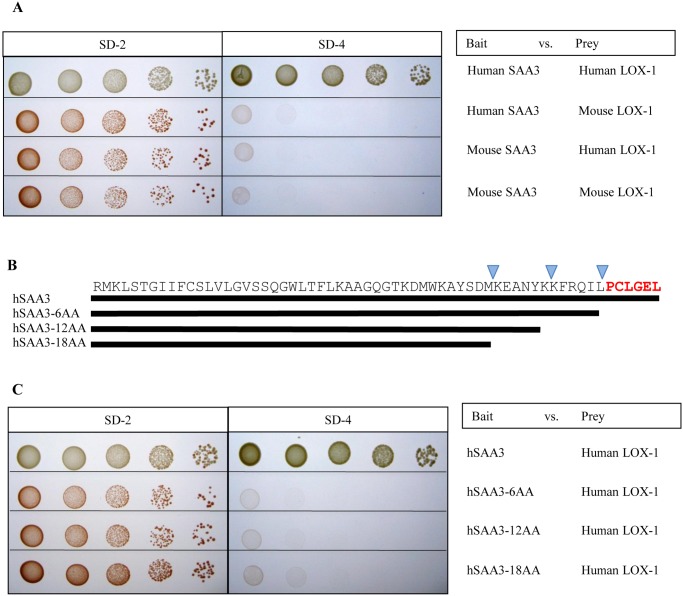
Yeast two-hybrid analysis for the interaction between SAA3 and LOX-1. (A) Bait constructs were the GAL4 DNA-binding domain fused to hSAA3 C-terminal 61 mer or mSAA3. Prey was the GAL4-activation domain fused to the human LOX-1 extracellular domain, or the mouse LOX-1 extracellular domain. The left panel shows the growth of transformed yeasts in synthetic dropout medium (SD-2; -Leu, -Trp). The middle panel indicates interactions between SAA3 and LOX-1 constructs (shown in the right panel) in SD-4 (-Leu, -Trp, -His, -Ade) in a series of dilutions (dilution increases from left to right). (B) Schematic representation of hSAA3 and its serial C-terminal deletion mutants. An actual amino acid sequence is also displayed. (C) Bait constructs were the GAL4 DNA-binding domain fused to hSAA3 C-terminal 61 mer (hSAA3), and its 6 (hSAA3–6AA), 12 (hSAA3–12AA), or 18 amino acid deletion mutant (hSAA3–18AA). Prey was the GAL4-activation domain fused to the human LOX-1 extracellular domain. The left panel shows the growth of transformed yeasts in the synthetic dropout medium (SD-2; -Leu, -Trp). The middle panel indicates interactions between SAA3 and LOX-1 constructs (shown in the right panel) in SD-4 (-Leu, -Trp, -His, -Ade) in a series of dilutions (dilution increases from left to right).

The yeast two hybrid assays were performed to search for a binding partner of hSAA3. The C-terminal 61 amino acids (hSAA2-SAA3a) were used as bait and human placenta cDNA library was used as prey for screenings. Our two hybrid assay revealed human LOX-1 to be a good candidate for a receptor of hSAA3 (Fig. 3A). A confirmation assay gave positive results between hSAA3 and human LOX-1, whereas no interaction was observed between mSAA3 and mouse or human LOX-1. To determine the binding site of hSAA3 against human LOX-1, serial deletion mutants were tested on the yeast two hybrid assays. The results showed that hSAA3 C-terminal 6 amino acids, unique to hSAA3, were necessary to bind human LOX-1 (Fig. 3B, C).

**Fig 4:**
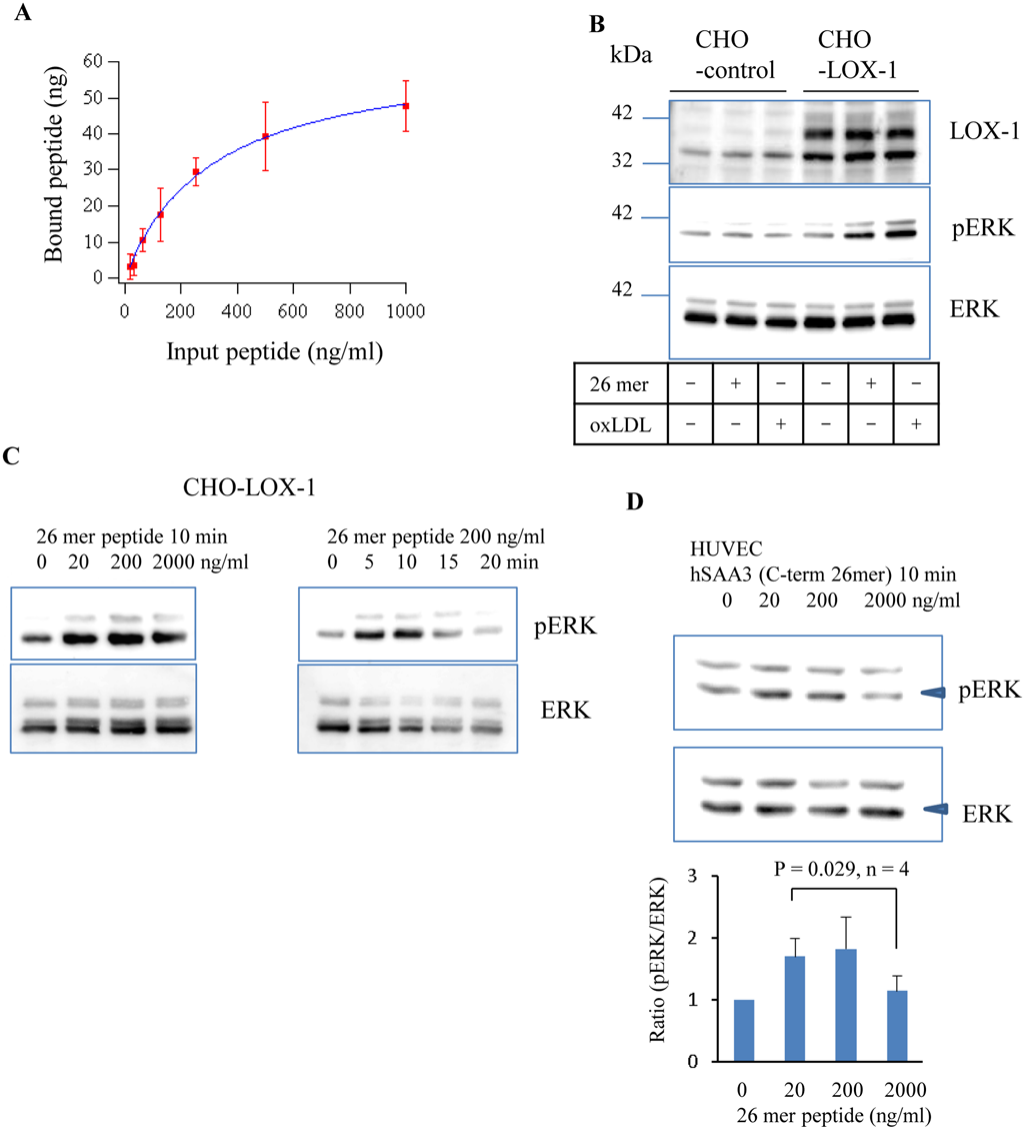
The hSAA3 C-terminal peptide binds LOX-1. (A) hSAA3 C-terminal 26 mer peptide input vs bound on human LOX-1. Unit, ng/ml. Error bars represent the standard deviation of four independent experiments. Dissociation constant of the synthetic peptide against human LOX-1 was determined as Kd = 99 ± 22 nM. (B) LOX-1, phospho-ERK, and ERK immunoblottings of the control CHO and human LOX-1 expressing CHO cell lysates 10 min after the hSAA3 C-terminal 26 mer peptide (200 ng/ml) or oxLDL (100 μg/ml) stimulation. (C) Phospho-ERK and ERK immunoblottings of LOX-1 expressing the CHO cell lysate with or without the hSAA3 C-terminal 26 mer peptide. (D) Phospho-ERK and ERK immunoblottings of HUVEC cell lysates with or without the hSAA3 C-terminal 26 mer peptide. Densitometry quantification (normalized by without peptide) of the bands marked by arrow heads is shown below (four independent experiments).

The *in vitro* binding assay determined the Kd value of the hSAA3 C-terminal 26 mer syntheticpeptide against the LOX-1 protein (Kd = 99 ± 22 nM) (Fig. 4A). A previous study reported that oxLDL binding on LOX-1 enhanced ERK protein phosphorylation [18]. In Fig. 4B, CHO cells with a human LOX-1 expression system responded to the hSAA3 C-terminal 26 mer peptide (200 ng/ml, 10 min) or oxLDL stimulations, which enhanced the phosphorylation of ERK, while control CHO cells did not. Fig. 4C presents the phosphor-ERK western blot analysis of CHO-LOX-1 cells 10 min after the hSAA3 C-terminal 26 mer peptide at various concentrations and 0, 5, 10, 15, or 20 min after stimulation with 200 ng/ml of hSAA3 C-terminal 26 mer peptide stimulation. HUVEC, expressing native LOX-1, stimulated with the hSAA3 C-terminal peptide for 10 min, were also analyzed (Fig. 4D). Thus, LOX-1-dependent intracellular signaling triggered by hSAA proteins can be similar to that by oxLDL.

**Fig 5:**
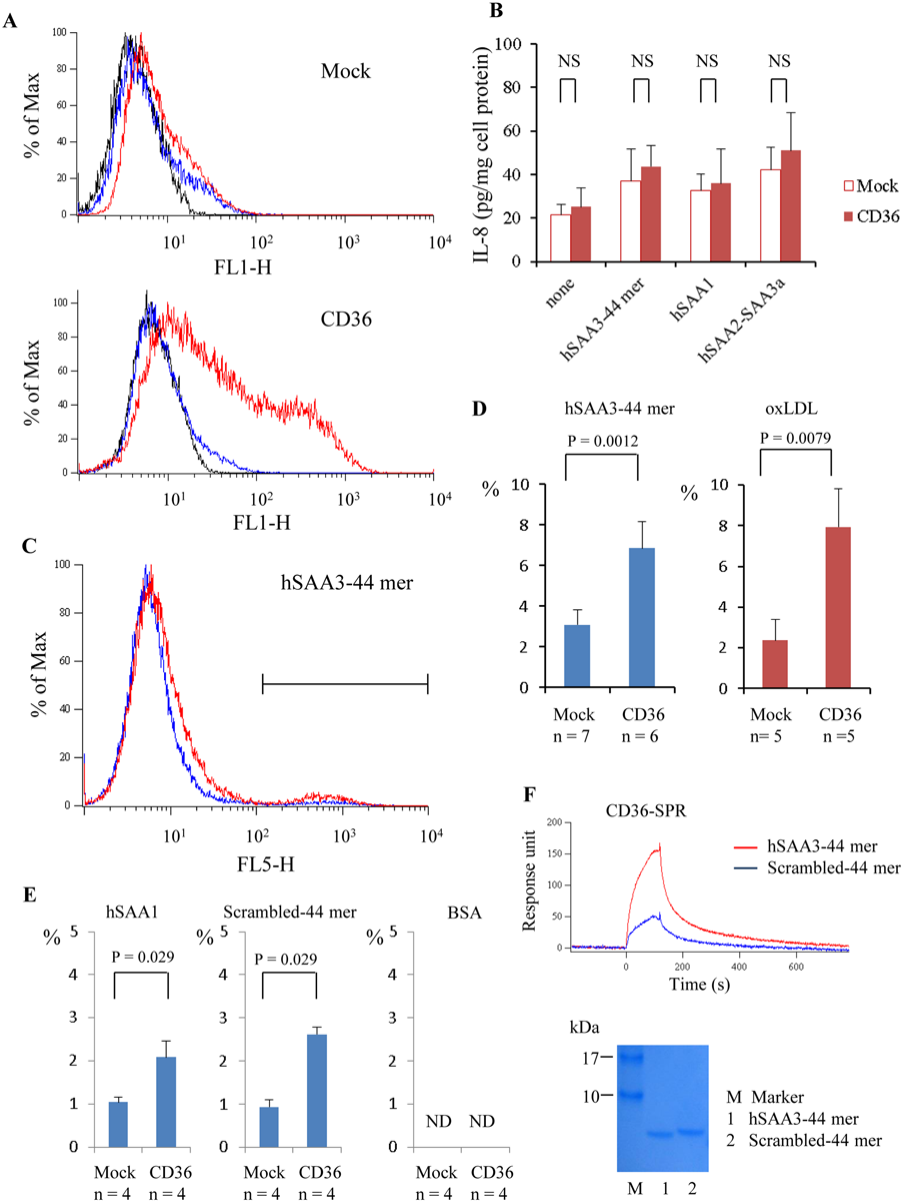
Interactions between hSAA3 C-terminal peptide and CD36. (A) Blank vector (top panel) or CD36 expression vector (bottom panel) transfected HEK293T cells were subjected to flow cytometry analysis. Solid, blue, and red lines represent histograms for no 1^st^ antibody, isotype control, and anti-CD36, respectively. (B) IL-8 levels in the culture supernatants. Blank vector or CD36 expression vector transfected HEK293T cells were incubated with hSAA1 (2 μg/ml), hSAA3 C-terminal 44 mer peptide (5 μg/ml), or hSAA2-SAA3a with DYKDDDDK tag (1 μg/ml) for 20 h in 0.2% BSA-DMEM. NS = not significant (P > 0.05). (C) HeLa cells were incubated with fluor-labeled hSAA3 C-terminal 44 mer (2 μg/ml) for 2 h in 0.2% BSA-DMEM. The cells were analyzed by a flow cytometer to estimate the ligand uptake. Blue and red lines represent histograms for mock and CD36 over-expressing HeLa cells, respectively (four independent experiments). (D) The ratios of the ligand uptake (fluor-labeled hSAA3–44 mer (2 μg/ml) or DiI-oxLDL (4 μg/ml)) indicated in the plot. (E) The rations of the fluor-labeled ligand (left) hSAA1 (2 μg/ml), (center) scrambled-44 mer peptide (2 μg/ml), and (right) BSA (2 μg/ml) are shown. ND = not detected. (F) Binding of hSAA3–44 mer peptide or scrambled-44 mer peptide on CD36 was analyzed by surface plasmon resonance. The peptide (10 μM, = 50 μg/ml) was applied (0–120 sec) on a sensor chip coated with recombinant CD36. Sensorgrams show association (0–120 sec) and dissociation (120–780 sec) of analytes (four independent experiments). The hSAA3–44 mer and scrambled-44 mer peptide were analyzed by SDS-PAGE. Analytes (100 ng each) were electrophoresed on a 15–20% gradient gel and stained with CBB.

It has been reported that CD36 functions as a receptor for SAA [15]. To investigate interactions between CD36 and hSAA3 domain, we performed IL-8 secretion assay. More than 50% of the CD36 over-expressing HEK293T cells were CD36 positive as revealed by flow cytometry analyses (Fig. 5A). Nevertheless, IL-8 secretion from CD36 over-expressing HEK293T cells was not drastically enhanced upon stimulation of hSAA3 44 mer peptide, hSAA1 recombinant protein, or hSAA2-SAA3 fusion protein (Fig. 5B). Next, we assessed fluor-ligand uptake of HeLa cells. The flow cytometry analysis of CD36 over-expressing HeLa cells yielded similar results shown in Fig. 5A. Fluor-labeled hSAA3 44 mer peptide or hSAA1, or DiI-oxLDL was applied onto control or CD36 over-expressing HeLa cells and the cells were subjected to flow cytometry analysis. The fluorescence positive cells displayed in the marked region in Fig. 5C were counted to calculate the ratio (Fig. 5D). The result indicates that hSAA3 C-terminal 44 mer peptide uptake was enhanced by CD36 expression, and similar results were observed in the case of oxLDL (Fig. 5D, right) and hSAA1 (Fig. 5E, left). Surprisingly, scrambled 44 mer were also endocytosed into HeLa cells in a CD36 dependent manner (Fig. 5E, center). On contrary, both mock and CD36 over-expressing HeLa cells failed to incorporate flour-labeled BSA (Fig. 5E, right). The scrambled 44 mer has same amino acid contents, but not amino acid sequence similarity against the hSAA3 44 mer. We further analyzed by a SPR to find out that both peptides were able to bind on CD36 immobilized on a sensor chip (Fig. 5F, top). The hSAA3 44 mer peptide gave higher response in the sensorgram than scrambled 44 mer, implying that the hSAA3 44 mer has higher binding affinity to CD36. SDS-PAGE analysis indicated that concentrations of peptides (5 kDa) used in the SPR analysis were almost same as each other (Fig. 5F, bottom).

## Discussion

Recent genome wide investigations have revealed that approximately 4–6% genes are expected to have transcription-induced chimeras, readthrough fusions across adjacent genes in the genome [19, 20]. Some fusion chimeras have been found in cancer tissues or cell lines, but not in non-tumor tissues [21–23], while other chimeras generate functionally distinctive gene products that may play an important role in cell differentiation [24]. However, the vast majority of readthroughs have not been examined in enough detail to identify their physiological functions or importance in biological events. The detection of transcription-induced chimeras is more difficult than canonical transcript quantification because their copy numbers are low and it shares the nucleotide sequence with the canonical transcript.

In our previous studies, mSAA3 was identified as a key molecule to build up the pre-metastatic phase in tumor-bearing model mouse studies [6, 7, 10]. The expression level of mSAA3 was maintained at a high level in the lungs of tumor-bearing mice and mSAA3 antibody therapy effectively blocked lung metastasis. High levels of mSAA3 expression upon stimulation with cytokines, such as TNFα, derived from the primary tumor site can only be seen in lung endothelial or epithelial cells, along with myeloid derived immune cells. This specificity accounts for the propensity towards lung metastasis; therefore, the SAA3 molecule is a good candidate for a target molecule for the diagnosis or treatment of lung metastasis. Human SAA3 is regarded as a pseudogene because it encodes a short open reading frame [12].

The nucleotide sequence of hSAA3 is very similar to that of mSAA3; however, because of a single nucleotide insertion, which is not seen in other mammalian genomes with a few exceptions, a frame shift occurred at hSAA3 exon 2 to make its deduced amino acid sequence completely different from that of the mouse protein. A stop codon existing shortly after the insertion site alters the reading frame to give it a total product size of 60 amino acids. Our 5’ RACE revealed, for the first time, that hSAA3 exon 1 or hSAA3 exon 2 is fused with hSAA2 exon 3. Human SAA2 was shown to have a readthrough with hSAA4, with sequence information being deposited in the UCSC genome database. In this case, hSAA2 exon 3 is attached to hSAA4 exon 2 to give a potential 208 amino acid peptide, which includes the entire hSAA4 peptide, although evidence has never been reported at the protein level. We confirmed that RT-PCR using the cDNAs used in this study as a template was able to amplify the hSAA2-SAA4 readthrough. It is noteworthy that the distance between hSAA2 exon 3 and hSAA3 exon 1 is approximately 130 kb, while hSAA4 exon 2 is located 10 kb downstream of hSAA2 exon 3. According to a genome wide search reporting an exon-exon gap for a fused gene larger than 200 kb, long distance exon-exon gap was rare [19]. The (hSAA2 exon 3)-(hSAA3 exon1) junction allows in-frame translation to generate a chimera protein with a total of 138 amino acids, consisting of SAA2 76 amino acids (residue 1–76), two amino acids (Ser, Arg) in hSAA3 exon 1, and the next 60 amino acids in hSAA3 exon 1 and exon 2, which has been described as the deduced amino acid sequence of hSAA3 in a previous study [16]. On the other hand, the (hSAA2 exon 3)-(hSAA3 exon 2) junction has a stop codon shortly after the junction. This produces truncated hSAA2 protein (hSAA2t) and very low levels of the second cistron, encoding hSAA3t. Although translation of the second cistron is generally possible, its translation level is markedly lower than that of the first cistron. Given the low expression level of hSAA2-SAA3b transcript, expression of the second cistron hSAA3t can be disregarded. Thus, we decided to focus on the hSAA2-SAA3 fusion protein.

We succeeded in capturing hSAA3 in immunoprecipitation-ELISA from T47D cell culture media and cell lysates, although we were not able to determine if the captured molecules were the hSAA2-SAA3 fusion protein or hSAA3 domain alone. Based on our qPCR and ELISA data, it is assumed that hSAA3 level in tissues is very low. So far, hSAA3 has not been reported in clinical samples. In many cases, the hSAA3 serum level might be much lower than the Kd value of hSAA3 binding to LOX-1 determined by our biochemical analysis (99 nM). In contrast, the SAA2 protein levels are high in human tissues, and these are markedly enhanced in the event of inflammation. Therefore, even though readthrough expression levels are commonly lower than those of the parental genes, it was not surprising to observe the hSAA2-SAA3 fusion protein. It is also possible that alternative splicing happens to form hSAA2-SAA3 fusion transcripts in particular cases.

T47D cell lines were considered to be non-metastatic cells as MCF7 [25, 26]. There has been no apparent correlation between hSAA3 expression and metastasis capability in human cell lines. It is attributed to the difference in the affinity of mSAA3 and hSAA3 against the TLR4/MD-2 complex. Mouse SAA3 bound TLR4/MD-2 to activate downstream inflammatory responses [11], whereas hSAA3 failed to bind TLR4/MD-2 (unpublished data). We have pointed out the importance of K44 residue in mSAA3 (amino acid sequence;-K43-K44-A45-N46-) for TLR4/MD-2 binding, and K44 does not exist in hSAAs including hSAA2-SAA3a [11]. The corresponding amino acid sequence (-KKAN-) is replaced with in hSAA2 (-REAN-), and hSAA3 (-KEAN-), respectively.

Our yeast two hybrid assay captured LOX-1 as a hSAA3 binding protein. LOX-1 holds a small cytoplasmic domain at the N-terminal region and a C-type lectin like domain at the C-terminal region. Although its physiological functions have yet to be elucidated, it is known to be important in the control of vascular dynamics. Several studies have reported that LOX-1 is involved in endothelial dysfunction and atherogenesis [27–31]. A recent study revealed that C-reactive protein, a novel inflammation marker protein, was a potential protein ligand for LOX-1 other than oxLDL, which provided some insight into identifying the new functions of both LOX-1 and C-reactive protein [32, 33]. The results of the present study indicate that LOX-1 can also recognize the hSAA2-SAA3 fusion protein. Its recognition site is the C-terminal of the fusion protein; therefore, the C-terminal hSAA3 peptide was enough to activate the receptor. This C-terminal region of the hSAA3 peptide is so unique that no other homologue is found in the human protein database, which suggests that the hSAA2-SAA3 fusion protein may possess physiological functions distinct from the other SAA molecules. Due to the technical difficulty for preparing highly purified hSAA2-SAA3 fusion protein, hSAA3 peptide (26 mer or 44 mer) were used in the *in vitro* assays. There might be possible that structure of hSAA3 C-terminal moiety in hSAA2-SAA3 fusion protein differs from that of synthetic peptides. If the C-terminal region is folded in the core of the protein, the unique peptide may not have a chance to interact with extracellular receptor such as LOX-1. However, SAA degradation is not aberrant; for instance, most amyloid A proteins have undergo proteolysis in amyloid A amyloidosis [34]. In that case, it is possible that detached unique peptide gains its function.

This study showed hSAA3-LOX-1 stimulation activated ERK to some extent. ERK activation generally promotes cell survival or proliferation [35]. It also mediates anti-proliferative events including apoptosis, autophagy and senescence [36, 37]. We were not able to determine which type of the effect (proliferation or anti-proliferation) was dominant in the case of hSAA3-LOX-1 signaling. Baranova et al. have shown that SAA binds CD36 to activate MAPK signaling including ERK phosphorylation [15]. In this study we demonstrated CD36 dependent hSAA3 C-terminal peptide uptake, suggesting that CD36 might have a role in hSAA3 signaling pathway. Moreover, the synthetic hSAA3 peptide was shown to exhibit unique biological activity [38, 39]. Thus, future research is needed to decipher the hSAA3 biological functions *in vivo* and *in vitro*.

In conclusion, this is the first study to unveil the existence of hSAA3 at a protein level, which had been considered to be a pseudogene without being translated into a polypeptide chain, and also suggested the possibility that the hSAA3 C-terminal region may function as a ligand for the LOX-1 receptor protein to enhance the phosphorylation of ERK proteins.

## Supporting Information

S1 Fig Ct value vs log(copy number) for qPCR primer/probes. (DOCX)

S2 Fig Q-Q plots (Experimental value vs Expected value) for the data shown in Fig. 2C. (DOCX)

S3 Fig Q-Q plots (Experimental value vs Expected value) for the data shown in Fig. 2D. (DOCX)

S1 Table RT-qPCR and RT-PCR primers tested in this study. (DOCX)

S2 Table Statistical hypothesis testing information for Fig. 2C showing relative hSAA2 copy number. (DOCX)

S3 Table Statistical hypothesis testing information for Fig. 2D showing relative hSAA3 copy number. (DOCX)
