# Supplementary figures and images for: Human Serum Amyloid A3 (SAA3) Protein, Expressed as a Fusion Protein with SAA2, Binds the Oxidized Low Density Lipoprotein Receptor
Source: PLoS One. 2015 Mar 4;10(3):e0118835. doi: 10.1371/journal.pone.0118835 (PMC4349446; doi:10.1371/journal.pone.0118835)

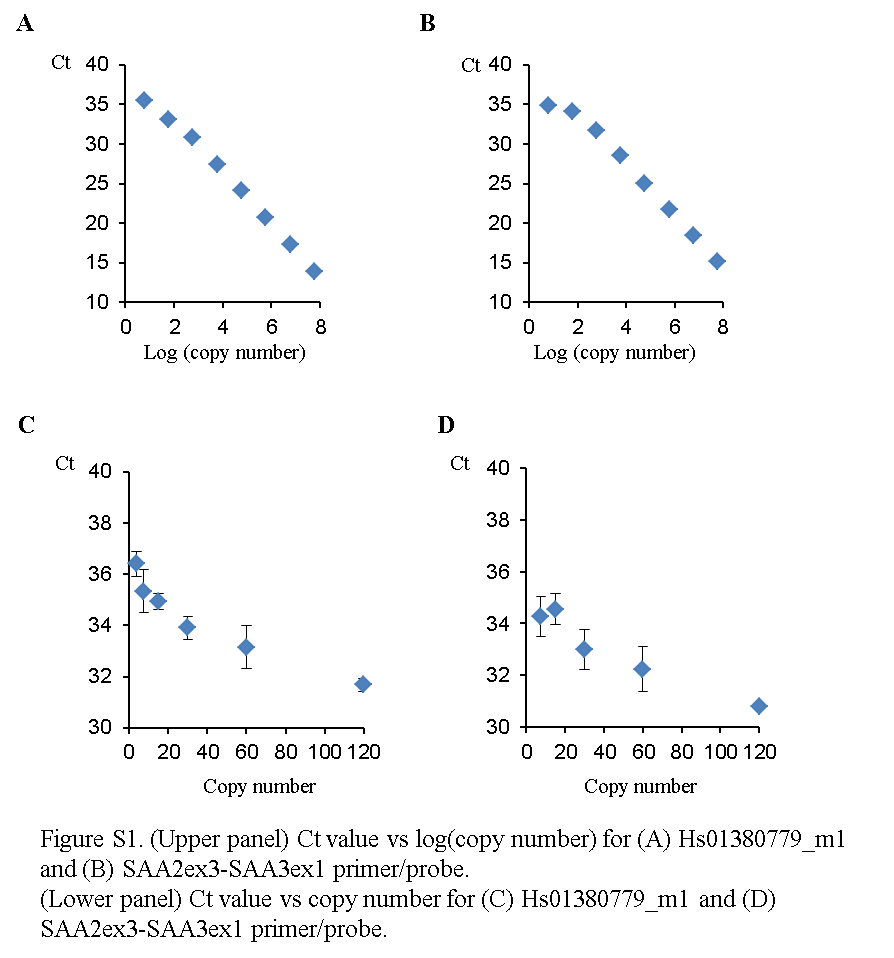

Supplement: S1 Fig — (DOCX) [file pone.0118835.s001.docx]
