# Supplementary material for: Human Serum Amyloid A3 (SAA3) Protein, Expressed as a Fusion Protein with SAA2, Binds the Oxidized Low Density Lipoprotein Receptor
Source: PLoS One. 2015 Mar 4;10(3):e0118835. doi: 10.1371/journal.pone.0118835 (PMC4349446; doi:10.1371/journal.pone.0118835)
Supplement: S2 Fig — (DOCX) [file pone.0118835.s002.docx]

**
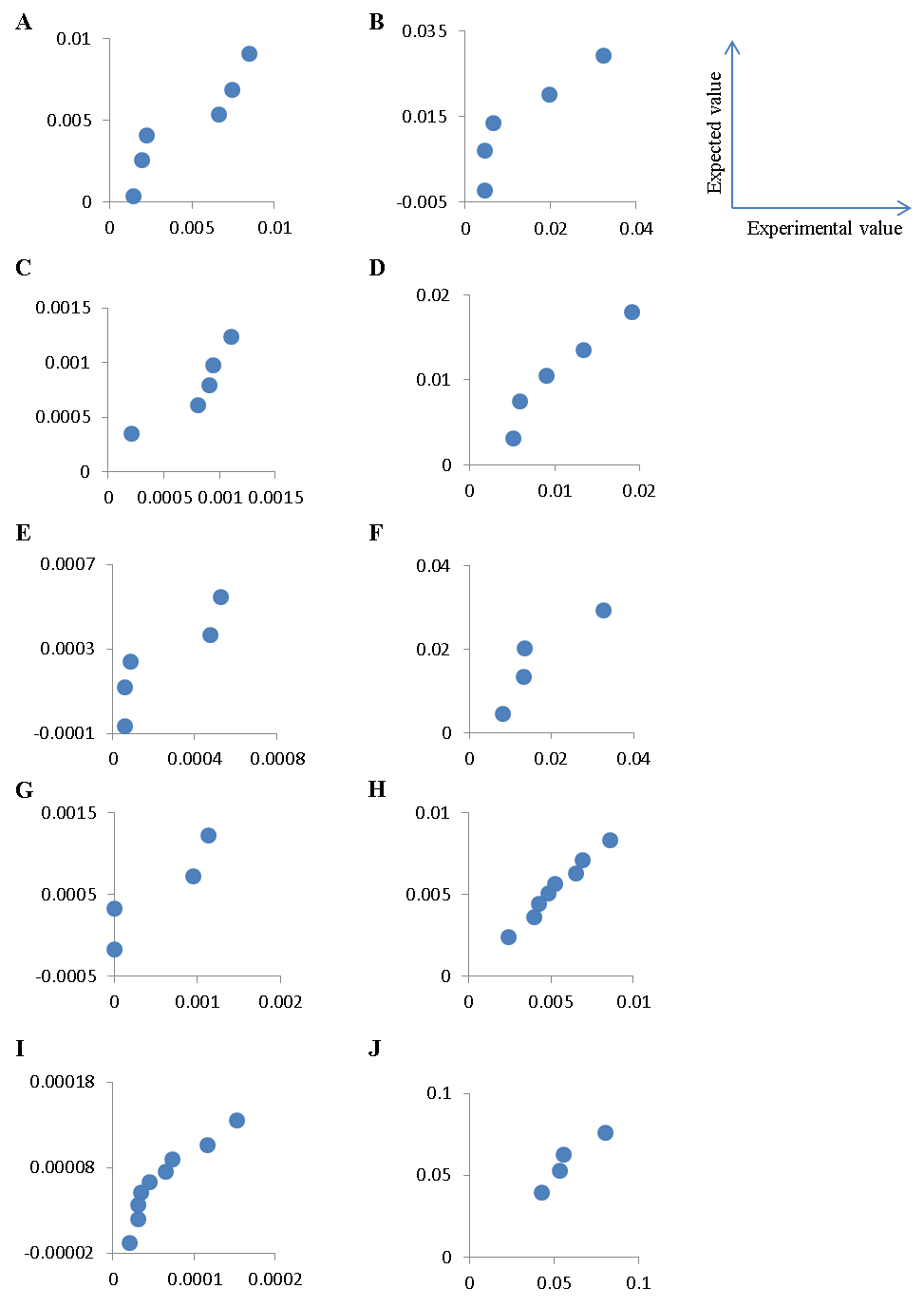
Figure S2**. Q-Q plots (Experimental value vs Expected value) for the data shown in Figure 2C. H292-control (A), H292-IL-1-IL-6-Dex (B), LU65-control (C), LU65-IL-1-IL-6-Dex (D), LU99-control (E), LU99-IL-1-IL-6-Dex (F), MCF7-control (G), MCF7-IL-1-IL-6-Dex (H), T47D-control (I), and T47D-IL-1-IL-6-Dex (J).
