# Supplementary material for: Human Serum Amyloid A3 (SAA3) Protein, Expressed as a Fusion Protein with SAA2, Binds the Oxidized Low Density Lipoprotein Receptor
Source: PLoS One. 2015 Mar 4;10(3):e0118835. doi: 10.1371/journal.pone.0118835 (PMC4349446; doi:10.1371/journal.pone.0118835)
Supplement: S3 Fig — (DOCX) [file pone.0118835.s003.docx]

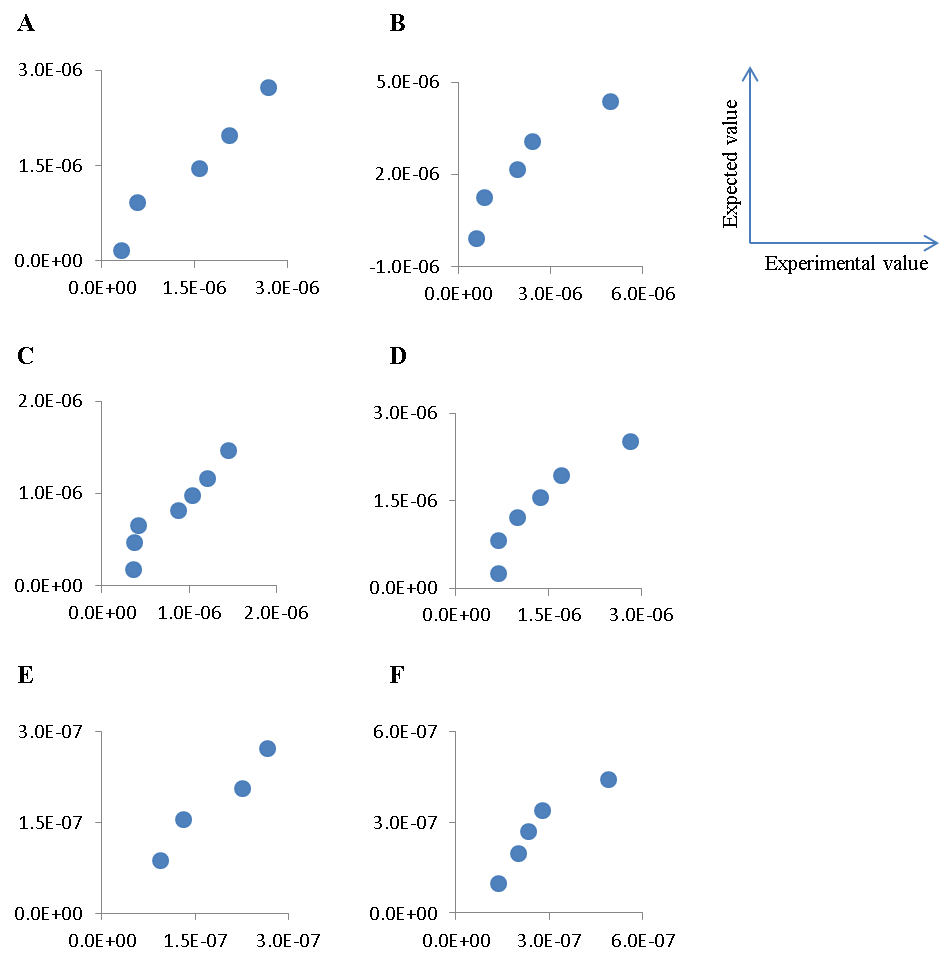


**Figure S3**. Q-Q plots (Experimental value vs Expected value) for the data shown in Figure 2D. LU65-control (A), LU65-IL-1-IL-6-Dex (B), LU99-control (C), LU99-IL-1-IL-6-Dex (D), T47D-control (E), and T47D-IL-1-IL-6-Dex (F).
