# Supplementary material for: Human Serum Amyloid A3 (SAA3) Protein, Expressed as a Fusion Protein with SAA2, Binds the Oxidized Low Density Lipoprotein Receptor
Source: PLoS One. 2015 Mar 4;10(3):e0118835. doi: 10.1371/journal.pone.0118835 (PMC4349446; doi:10.1371/journal.pone.0118835)
Supplement: S1 Table — (DOCX) [file pone.0118835.s004.docx]

**Table S1.** RT-qPCR and RT-PCR primers tested in this study

Gene specific primer used for 1st strand synthesis

GSP#2 5’-TTCAGTAACTGTAGGTCAAC-3’

GSP#8 5’-TGGATTTCACAAGCATTTATTAAGC-3’

PCR primers

SAA2ex2F#1 5’-GCCTACTCTGACATGAGAGAAGCCAA-3’

SAA3ex1F#1 5’-ATGAAGCTCTCCACTGGCATCATTTTC-3’

SAA3ex2R#1 5’-ATTACCTGATCACTTCTGTAGCCCAGACAG-3’

SAA3ex2R#2 5’-GCTCTGCTCACTCATTCCTGGCAACA-3’

SAA3ex2R#3 5’-GCAATCCTCTGCATGGTCTCCTGTGA-3’

By using these primer sets, PCRs were performed and the products were subjected to an agarose gel electrophoresis. The resulting bands were purified and their sequences were determined. Based on sequencing results we concluded that major PCR product from oligo dT primer or random primer derived cDNA were not SAA3 related. In contrast, gene specific primer derived cDNA generated SAA3 containing transcripts as main products. We used these two (GSP#2 and GSP#8) gene specific primers. For PCR reactions, SAA3ex1F#1-SAA3ex2R#1 primer set was best in terms of sensitivity and selectivity to amplify SAA3. The SAA2ex2F#1-SAA3ex2R#2 primer set was used to isolate SAA2-SAA3 transcript. In this case SAA3ex2R#2 was better than others.

qPCR primer/probes

SAA3ex1-SAA3ex2; Hs01380779_m1 (Life Technologies)

SAA3ex1; forward primer 5’-CCCTGGTCCTGGGTGTCA-3’

probe 5’-CCAAGGATGGTTAACATT-3’

reverse primer 5’-CTTGGCCAGCTGCCTTGA-3’

SAA2ex3-SAA3ex1; forward primer 5’-TGTGGAGAGCCTACTCTGACATGA-3’

probe 5’-AGGATGAAGCTCTCCACTGGCATCAT-3’

reverse primer 5’-TTAACCATCCTTGGCTGCTGACAC-3’

SAA2ex3-SAA3ex2; forward primer 5’-CCGCAGAAGTGATCAGGGACTAAA-3’

probe 5’-TGTCTGGGCTACAGAAGTGATCAGGT-3’

reverse primer 5’-CTGCTCACTCATTCCTGGCAACAT-3’

Among these primer/probe sets, Hs01380779_m1 was the best in terms of sensitivity and data linearity. Plasmid containing the SAA2-SAA3 readthrough was diluted to make standard solutions. Quantitative PCR reactions were carried out by using these standard solutions as templates. Logarithms of the copy number of each template were plotted against its Ct value determined by the qPCR. Result for Hs01380779_m1 is shown in Figure S1A and S1C. This has better linearity in the low copy number region than other primer set shown in Figure S1B and S1D (Error bars represent standard deviation from four independent measurements). It should be noted that the SAA3 copy numbers quantitated in this study were smaller than 100 in all cases.
