# Supplementary material for: Human Serum Amyloid A3 (SAA3) Protein, Expressed as a Fusion Protein with SAA2, Binds the Oxidized Low Density Lipoprotein Receptor
Source: PLoS One. 2015 Mar 4;10(3):e0118835. doi: 10.1371/journal.pone.0118835 (PMC4349446; doi:10.1371/journal.pone.0118835)
Supplement: S2 Table — (DOCX) [file pone.0118835.s005.docx]

**
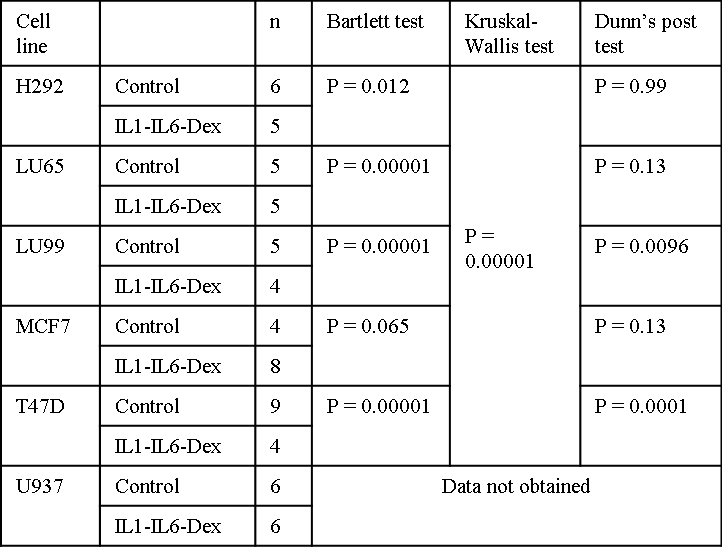
Table S2.** Statistical hypothesis testing information for Figure 2C showing relative hSAA2 copy number.

In order to check the normality, we performed Shapiro-Wilk tests for the data sets (Figure 2C, 2D, 2F, 4A, 4D, 5B, and 5D). For Figure 2C and 2D data the null hypothesis was rejected, while in other cases it was accepted. Figure 2F, 4A, 4D, 5B, and 5B data were analyzed using Welch’s t-test, and Mann-Whitney U-test gave same conclusions. Next, Bartlett test was performed to test the equality of two variances for the latter data sets, and Q-Q plot for each data set was prepared to visualize the pattern in the data. And then, Figure 2C and 2D data were analyzed by Kruskal-Wallis test and Dunn’s post test. Table S2 and Figure S2A-J present the results for Figure 2C, and Table S3 and Figure S3A-F for Figure 2D.
