# Supplementary material for: Human Serum Amyloid A3 (SAA3) Protein, Expressed as a Fusion Protein with SAA2, Binds the Oxidized Low Density Lipoprotein Receptor
Source: PLoS One. 2015 Mar 4;10(3):e0118835. doi: 10.1371/journal.pone.0118835 (PMC4349446; doi:10.1371/journal.pone.0118835)

**Table S3.** Statistical hypothesis testing information for Figure 2D showing relative hSAA3 copy number.


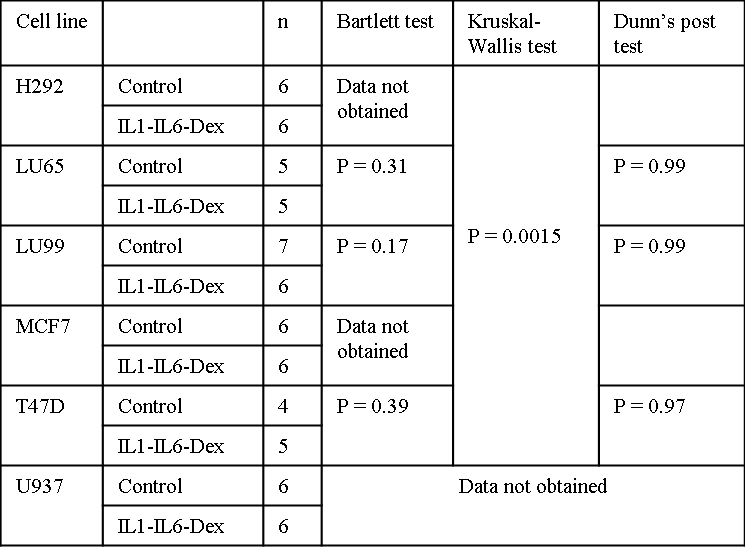

Supplement: S3 Table — (DOCX) [file pone.0118835.s006.docx]
